# Evaluation of Intestinal Parasite Infection in Low and High Coverage of Graduated Households, Northwest Ethiopia: A Comparative-Based Crosssectional Study

**DOI:** 10.1155/2021/6651100

**Published:** 2021-05-11

**Authors:** Desalegn Andargie, Yalewayker Tegegne, Ligabaw Worku

**Affiliations:** ^1^University of Gondar Comprehensive Specialized Hospital Laboratory, College of Medicine and Health Sciences, University of Gondar, Gondar, Ethiopia; ^2^Department of Medical Parasitology, School of Biomedical and Laboratory Sciences, College of Medicine and Health Sciences, University of Gondar, Gondar, Ethiopia

## Abstract

Intestinal parasite infections are widely distributed and affect various segments of the population in Ethiopia as in many developing countries. The government launched an innovative program called Health Extension Program to increase the coverage of primary health care services, mainly by producing model households using model-family training. The aim of this study was to evaluate the intestinal parasite infection in low and high coverage of graduated households. *Method*. A community-based crosssectional study was conducted from February to June, 2019. A total of 478 participants were enrolled in this study by using a multistage sampling technique. Data were collected by using pretested and semistructured questionnaire. Five grams of stool specimen was collected, and samples were processed using a direct wet mount and Kato Katz technique. Data were coded, entered, and cleaned using statistical package for social science (SPSS) version 20. A Chi-square test was employed to compare the two groups. *P* value < 0.05 were taken as statistically significant. *Result*. The prevalence rate of IPIs was 39% and 20.5% in LCGHH and HCGHH, respectively. *A. lumbricoides* was the predominant parasite, detected in 14.6% and 8.8% followed by *S. mansoni* 6.3% and 2.1% in LCGHH and HCGHH districts, respectively. LCGHH had significantly higher prevalence of *A. lumbricoides*, *S. mansoni*, and hookworm infections than the HCGHH district (*P* < 0.05). Thirteen (18.8%) study participants in LCGHH and four (11.7%) in HCGHH showed heavy infection with the four common soil-transmitted helminths (*A. lumbricoides*, *S. mansoni*, hookworm, and *T. trichiura*). Among study participants who were positive for *S. mansoni*, 53.3% in LCGHH and 20% in HCGHH had heavy infection for the Kato thick smear used. *Conclusion*. The prevalence of IPIs is significantly higher in LCGHH than in the HCGHH district. Producing more model households by giving model family training to nonmodel households and strengthening the information, education, and communication package are crucial in the implementation of the HEP to decrease the prevalence of IPIs especially in LCGHH districts.

## 1. Introduction

Intestinal parasites are among the common health problem of world population and significantly higher in developing countries [1]. This is due to socioeconomic status of the countries and environmental, ecological, and behavioral factors particularly in the Sub-Saharan Africa.

Intestinal parasite infections are widely distributed and affect various segments of the population in Ethiopia as in many developing countries [2]. The prevalence estimated that one-third, one-quarter, and one in eight of the population are infected with *Ascaris lumbricoides*, *Trichuris trichiura*, and Hookworm, respectively. As a result, Ethiopia has the second highest burden of ascariasis, the third highest burden of hookworm infection, and the fourth highest burden of trichuriasis in Sub-Saharan Africa [3].

In 2003, the Ethiopian government launched a Health Extension Program (HEP) to provide basic health care through the use of trained Health Extension Workers (HEWs). The HEP has 16 packages, among these 6 packages are under hygiene and environmental sanitation (excreta disposal, soil and liquid waste disposal, water supply and safety measures, food hygiene and safety measure, healthy home environment, and personal hygiene) which are targeted to reduce intestinal parasite (IP) infections [4, 5].

The HEP is designed to increase the coverage of primary health care services (PHCS), mainly by producing model households using model family training. The model family training comprises a total of 96 hours of training on basic hygiene and environmental sanitation (30 hours), family health care (42 hours), and disease prevention and control (24 hours). Households which attend at least 75% of the training and implement at least 75% of the HEP packages receive certificates and graduate as model households (families) [6–9]. Currently, there are high and low coverage of graduate household in the same and/or different district with IP infection. However, assessment of IP infection would facilitate further progress for HEP. Therefore, the aim of this study was to evaluate IP infection in low and high coverage of graduated households (LCGHH and HCGHH).

## 2. Main Text

### 2.1. Materials and Methods

#### 2.1.1. Study Design, Period, and Area

A community-based crosssectional study was conducted in West Denbiya and Gondar Zuriya districts, Central Gondar zone, Amhara region, Northwest Ethiopia, from February to June, 2019. West Denbiya district is located 62 Km from Gondar town and 789 Km from Addis Ababa, northwest part of Ethiopia. An estimated total population of west Denbiya is 133,875 and with 31,134 households in 24 Kebeles, and (14%) the households were graduated in this district where the average temperature is 28°C. The second district with a HCGHHs in which (77%) households graduated is Gondar Zuriya, which is located 41 Km from Gondar town and 686 Km from Addis Ababa, northwest part of Ethiopia. An estimated total population is 239,080 and with 55,600 households in 44 Kebeles of Gondar Zuriya district.

#### 2.1.2. Sample Size and Sampling Technique

A multistage sampling technique was used to select samples from two districts (encompassing a number of kebele). From all kebeles (clusters) in each district (24 kebeles from LCGHHs and 44 kebeles from HCGHHs), three kebeles were selected by simple random sampling technique. The unit of study for this study was the household. To determine the number of households to be included in the study, a single population proportion formula for sample size calculation was used. The minimum sample size was calculated using single population proportion formula (Z*α*/2)^2^(*p*) (1 − *p*)/*d*^2^, with the following assumption: prevalence (*p*) of 83% from the previous study (), 95% confidence level, and 5% margin of error. Accordingly, the minimum sample size (*n*) including ten percent (10%) nonresponse rate was 478.

#### 2.1.3. Data Collection

Socio-demographic data and environmental and behavioral factors were collected by using pretested and standardized questionnaire by health officers through interview, which was translated into the local language Amharic.

#### 2.1.4. Stool Sample Collection

Each study participants were advised to bring about 5 gms of fresh stool and was given a labeled, clean, dry, and leak proof stool cup. Direct saline and Kato thick smear were processed inside the temporary laboratory established at the site collection. The intensity of infection was classified according to the guideline of WHO, for *S. mansoni*, light infection (1-99 epg), moderate (100-399 epg), and heavy (greater than 400 epg). Similarly, the classifications are *A. lumbricoides*: light infection (1-4999 epg), moderate (5000-49999 epg), and heavy (greater than 50,000 epg); *T. trichiura*: light infection (1-999 epg), moderate (1000-9999 epg), and heavy (greater than 10,000 epg); and for hookworm: light infection (1-1999epg), moderate (2000-3999 epg), and heavy (greater than 4,000 epg), where epg stands for the number of eggs per gram of stool [10].

#### 2.1.5. Data Quality Control

All necessary reagents, chemicals, and the performance of kits were checked by known positive and negative sample before processing and examination of samples of the study subjects. The data were checked for completeness, and any incomplete or misfiled questionnaires were recorrected under supervision. All slides were examined twice by two microscopist independently. The result of laboratory examination was recorded on well-prepared format carefully for confirmation of the result.

#### 2.1.6. Data Analysis and Interpretation

Data were coded, entered, and cleaned using statistical package for social science (SPSS) version 20. Data cleaning was performed to check for accuracy and consistency and missing values. Frequency tables were used to present the summarized data. A Chi-square test was employed to compare the two groups. *P* value less than 0.05 were taken as statistically significant.

#### 2.1.7. Ethical Consideration and Consent to Participate

Ethical clearance was obtained from the Research and Ethical Review Committee of School of Biomedical and Laboratory Sciences, College of Medicine and Health Sciences, University of Gondar. Moreover, a letter of support was secured from the leaders of each kebele administrators, district health office, and zonal health office. Informed verbal consent was also obtained from each study participants. Positive study participants were treated with appropriate drugs for free by linking them to the health centers.

## 3. Results

### 3.1. General Characteristics of Study Participants

A total of 478 study participants who live in districts with LCGHHs and HCGHHs of West Denbiya and Gondar Zuriya from February to May 2019 were included in the study. The total number of study participants in each group was 239. The mean age of the study subjects was 30 ± 17.2 years with a minimum and a maximum age of 1 year and 93 years, respectively. The proportion of female in LCGHHs and HCGHHs were 51.9% (124) and 52.7% (126), respectively. The majority of study participants were orthodox 81.6% (390), living in rural area 60.7% (290), illiterates 49.8% (238), have less than 5 family members 69.1% (330), and farmers 34.3% (164) (Table 1).

### 3.2. Distribution of Intestinal Parasites in Low and High Coverage of Graduated Households

Among the 478 study participants, 29.5% (141) were found to be infected with intestinal parasite. From 239 in each group, 39% (93) and 20.5% (49) study participants were positive for at least one species of intestinal parasite in LCGHH and HCGHH districts, respectively. *A. lumbricoides* was the predominant parasite, detected 14.6% in LCGHH and 8.8% in HCGHH district, and followed by *S. mansoni* 6.3% and 2.1% and hookworm 4.6% and 1.2% in LCGHH and HCGHH districts, respectively (Table 2).

**Figure 1:**
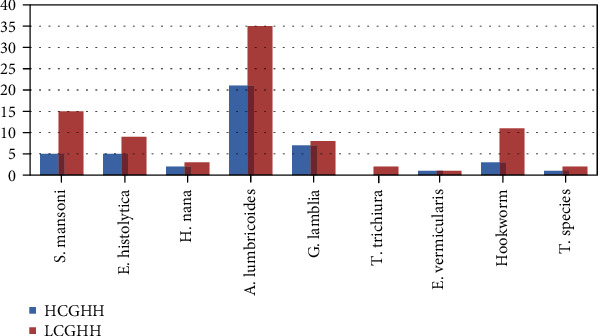
Frequency distribution of intestinal parasites.

Among protozoan parasite, *E. histolytica/dispar* was detected 3.8% (9) in LCGHH and 2.1% (5) in HCGHH district, while *G. lamblia* accounts 3.3% (8) in LCGHH and 2.9% (7) in HCGHH district of the study participants (Figure 1).

### 3.3. Prevalence of Intestinal Parasite in Low Coverage Compared to High Coverage of Graduated Households

The overall prevalence of IPIs in LCGHH and HCGHH districts was 39% and 20.5%, respectively. The difference in the prevalence of IPIs between the two districts were statistically significant (*P* value < 0.001). Among identified parasites, *A. lumbricoides*, *S. mansoni*, and hookworm were statistically significant associated between LCGHH and HCGHH. But there was no statistically significant association in *E. histolytica/dispar and G. lamblia* infection within the LCGHH and HCGHH districts (Table 2).

### 3.4. Intensity of Intestinal Helminth Infection

From intestinal helminths, infected study participants 18.8% in LCGHH and 11.7% in HCGHH showed heavy infection. From study participants who were positive for *S. mansoni*, 53.3% (8) in LCGHH and 20% (1) had heavy infection in HCGHH. Seventy-one point four percent (25%) and 52.4% (11) of *A. lumbricoides* infected participants had low infection in LCGHH and HCGHH, respectively, for the Kato thick smear used (Table 3).

## 4. Discussion

Knowledge on the existence and distribution of intestinal parasitic infections in a given community is a prerequisite for strength of planning and evaluating intervention programs. The present study also evaluates intestinal parasite prevalence, and intensity of infection in low and high coverage of graduated households among individual living in West Denbiya and Gondar Zuriya districts.

The demonstration that there was high prevalence of intestinal parasitic infection 29.5% in this study was consistent with the study conducted in Saudi Arabia, 32.2% [11]. For, instance, it is lower than a study done in Malaysia, 41% [12], in South Chennai India, 75.8% [13], Jimma 83% [14], and East Wollega 64.9% [15]. On the other hand, the prevalence of intestine parasite infections in the present study was higher than the study conducted in the South of Tehran, Iran, 10.7% [16]. The most plausible explanation for such differences might be due to the difference in culture, socio-economic status, and the difference in studied populations in the different studies.

In our study, the prevalence of IP infections in LCGHH 39% (93) was higher than HCGHH 20.5% (47). Similarly, we found higher prevalence of *A. lumbricoides* 14.6% (35), *S. mansoni* 6.3% (15), hookworm 4.6% (11), *H. nana* 1.2% (3), and *Teania* spp. 0.8% (2) in LCGHH than that in HCGHH district of *A. lumbricoides* 8.8% (21), *S. mansoni* 2.1% (5), hookworm 4.6% (11), *H. nana* 1.2% (3), and *Teania* spp. 0.4% (1). Moreover, the prevalence of intestinal protozoan parasite such as *E. histolytica/dispar* 3.8% (9) and *G. lamblia* 3.3% (8) was slightly higher in LCGHH compared with their prevalence in HCGHH (*E. histolytica/dispar* 2.1% (5) and *G. lamblia* 2.9% (7)).

This difference in the prevalence of intestine parasite infections between the two districts might be due to less practice of personal hygiene and environmental sanitation, source of drinking water, habit of wearing shoes, and awareness about health in LCGHH than HCGHH, and implementation of HEP in LCGHH 14% is much lower than that in HCGHH district 77%. This means that the majority of the population has less access to practice primary health care and simple health promoting factors in LCGHH compared to HCGHH.

In the current study, the prevalence of IP infections in LCGHH was higher 39% (93), compared with HCGHH 20.5% (47). The difference in prevalence of intestine parasite infections showed a statistically significant association between LCGHH and HCGHH (*P* < 0.001). The result of this finding was supported by a study conducted in Malaysia [17]. The prevalence of *A. lumbricoides*, *S. mansoni*, and hookworm in LCGHH was also higher than in HCGHH district and showed statistically significant association (*P* < 0.05). But there was no statistically significant association between protozoan parasites such as *E. histolytica/dispar* and *G. lamblia* infection within LCGHH and HCGHH districts. One possible reason for the high prevalence of IP seen in the LCGHH might be due to their residential area sanitation condition which exposes them to IP infections.

Evaluation of parasitic load of helminths is very important to estimate the effects of the parasite in a given community. In the current study, the intensity of soil-transmitted helminth infection has shown that majority of the cases had light to moderate infection which is in line with the result of a study conducted in Central America [18, 19]. This difference in intensity of infection has been observed that this might be due to the mass drug chemotherapy and people exposure to infection which may result to buildup of acquired immunity. And also, among 5 study participants who were positive for *S. mansoni*, 20% (1) and from 15 study participants 53.3% (8) of them had heavy infection in HCGHH and LCGHH, respectively. This variation might be due to lack of knowledge how to prevent and control intestinal helminth infection and practices on personal hygiene and environmental sanitation in LCGHH district than in HCGHH which are important to reduce intestinal parasite infections.

## 5. Limitations

The study was a crosssectional design, which does not allow for the establishment of causal relationships.

## Figures and Tables

**Table 1:** Socio-demographic characteristics of study participants in low and high coverage of graduated household districts, Northwest Ethiopia, 2019.

Variables		Frequency		
		LCGHH	HCGHH	Total
		% (*n*)	% (*n*)	% (*n*)
Residence	Urban	43.1 (103)	35.6 (85)	39.3 (188)
	Rural	56.9 (136)	64.4 (154)	60.7 (290)
Sex	Male	48.1 (115)	47.3 (113)	47.7 (228)
	Female	51.9 (124)	52.7 (126)	52.3 (250)
Age in years	<10 years	11.3 (27)	13.4 (32)	12.3 (59)
	11-20 years	22.6 (54)	21.3 (51)	22.0 (105)
	21-30 years	25.1 (60)	22.6 (54)	23.9 (114)
	31-40 years	23.8 (57)	19.2 (46)	21.3 (103)
	≥41 years	17.2 (41)	23.4 (56)	20.3 (97)
Religion	Orthodox	82.4 (197)	80.8 (193)	81.6 (390)
	Muslim	17.2 (41)	17.2 (41)	17.2 (82)
	Protestant	0.4 (1)	2.0 (5)	1.2 (6)
Family size	<5 members	71.2 (170)	66.5 (159)	68.9 (329)
	5-10 members	28 (67)	33.1 (79)	30.5 (146)
	>10 members	0.8 (2)	0.4 (1)	0.6 (3)
Educational status	Illiterate	47.3 (113)	52.3 (125)	49.8 (238)
	Primary school	23 (55)	15.1 (36)	19.0 (91)
	Secondary school	19.2 (46)	24.7 (59)	22.0 (105)
	Certificate and above	10.5 (25)	7.9 (19)	9.2 (44)
Occupation	Student	33.5 (80)	28.5 (68)	31.0 (148)
	Gov't employee	8.8 (21)	8.8 (21)	8.8 (42)
	Daily labor	4.2 (10)	5.0 (12)	4. (22)
	Farmer	35.1 (84)	33.5 (80)	34.3 (164)
	Merchant	9.6 (23)	7.1 (17)	8.4 (40)
	Others	8.8 (21)	17.2 (41)	12.9 (62)
Total		50 (239)	50 (239)	100 (478)

**Table 2:** Prevalence of intestinal parasite infections in low and high coverage of graduated households' districts, Northwest Ethiopia 2019.

Characters		LCGHH % (*n*)	HCGHH % (*n*)	*χ* ^2^	*P* value
IP status	Positive	39% (93)	20.5% (47)	18.600	<.001^∗∗^
	Negative	61% (147)	79.5% (190)		
*A. lumbricoides*	Positive	15% (35)	8.8% (21)	3.964	0.046^∗^
	Negative	85% (204)	91.2% (218)		
*S. mansoni*	Positive	6.3% (15)	2% (5)	5.220	0.022^∗^
	Negative	93.7% (224)	98% (234)		
Hookworm	Positive	4.6% (11)	1.2% (3)	4.709	0.030^∗^
	Negative	94.4% (228)	98.8% (236)		
*G. lamblia*	Positive	3% (7)	3.3% (8)	0.079	0.779
	Negative	97% (232)	96.7% (231)		
*E. histolytica/dispar*	Positive	3.8% (9)	2% (5)	1.177	0.278
	Negative	96.2% (230)	98% (234)		

**Table 3:** Intensity of helminth infection in low and high coverage of graduated households' districts with intestinal parasite infections Northwest Ethiopia, 2019.

IP species	LCGHH				HCGHH			
	Low % (*n*)	Moderate % (*n*)	High % (*n*)	Total % (*n*)	Low % (*n*)	Moderate % (*n*)	High % (*n*)	Total % (*n*)
*A. lumbricoides*	71.4 (25)	17.1 (6)	11.5 (4)	100 (35)	52.4 (11)	33.3 (7)	14.3 (3)	100 (21)
*S. mansoni*	20 (3)	26.7 (4)	53.3 (8)	100 (15)	60 (3)	20 (1)	20 (1)	100 (5)
Hookworm	45.5 (5)	54.5 (6)	0	100 (11)	33.3 (1)	66.7 (2)	0	100 (3)
*T. trichiura*	0	100 (1)	0	100 (1)	0	0	0	0
Total	49.2 (34)	32 (22)	18.8 (13)	100 (69)	53 (18)	35.3 (12)	11.7 (4)	100 (34)

## Data Availability

I confirmed that all the data for this manuscript are available; if someone wants to request the data, you can contact all authors.

## References

[1] Pullan R. L., Smith J. L., Jasrasaria R., Brooker S. J. (2014). Global numbers of infection and disease burden of soil transmitted helminth infections in 2010. *Parasites & Vectors*.

[2] Legesse M., Erko B. (2004). Prevalence of intestinal parasites among schoolchildren in a rural area close to the southeast of Lake Langano, Ethiopia. *Ethiopian Journal of Health Development*.

[3] Deribe K., Meribo K., Gebre T. (2012). The burden of neglected tropical diseases in Ethiopia, and opportunities for integrated control and elimination. *Parasites & Vectors*.

[4] Federal Ministry of Health (2007). *Health Extension Program in Ethiopia Profile, Health Extension and Education Center*.

[5] Federal Ministry of Health (2007). *Health Extension Program Implementations Guide Line*.

[6] Teklehaimanot H. D., Teklehaimanot A. (2013). Human resource development for a community-based health extension program: a case study from Ethiopia. *Human Resources for Health*.

[7] Bilal N. K., Herbst C. H., Zhao F., Soucat A., Lemiere C. (2011). *Health extension workers in Ethiopia: improved access and coverage for the rural poor*.

[8] Wang H., Tesfaye R., GNV R., Chekagn C. T. (2016). *Ethiopia Health Extension Program: An Institutionalized Community Approach for Universal Health Coverage*.

[9] Gebreegziabher E. A., Astawesegn F. H., Anjulo A. A., Kerie M. W. (2017). Urban health extension services utilization in Bishoftu town, Oromia regional state, Central Ethiopia. *BMC Health Services Research*.

[10] Montresor A., Crompton D. W. T., Hall A., Bundy D. A. P., Savioli L. (1998). *Guidelines for the evaluation of soil-transmitted helminthiasis and schistosomiasis at community level*.

[11] Al-Shammari S., Khoja T., El-Khwasky F., Gad A. (2001). Intestinal parasitic diseases in Riyadh, Saudi Arabia: prevalence, sociodemographic and environmental associates. *Tropical Medicine & International Health*.

[12] Sagin D., Mohamed M., Ismail G., Jok J., Lim L., Pui J. (2002). Intestinal parasitic infection among five interior communities at upper Rejang River, Sarawak, Malaysia. *The Southeast Asian Journal of Tropical Medicine and Public Health*.

[13] Dhanabal J., Selvadoss P. P., Muthuswamy K. (2014). Comparative study of the prevalence of intestinal parasites in low socioeconomic areas from South Chennai, India. *Journal of Parasitology Research*.

[14] Mengistu A., Gebre-Selassie S., Kassa T. (2007). Prevalence of intestinal parasitic infections among urban dwellers in southwest Ethiopia. *The Ethiopian Journal of Health Development*.

[15] Dori G., Tullu K., Ali I., Hirko A., Mekuria G. (2011). Prevalence of hookworm infection and its association with anemia among patients visiting Fenan Medical Center, East Wollega Zone, Ethiopia. *Ethiopian Medical Journal*.

[16] Arani A. S., Alaghehbandan R., Akhlaghi L., Shahi M., Lari A. R. (2008). Prevalence of intestinal parasites in a population in south of Tehran, Iran. *Revista do Instituto de Medicina Tropical de São Paulo.*.

[17] Ngui R., Ishak S., Chuen C. S., Mahmud R., Lim Y. A. (2011). Prevalence and risk factors of intestinal parasitism in rural and remote West Malaysia. *PLoS Neglected Tropical Diseases*.

[18] Smith H., DeKaminsky R., Niwas S., Soto R., Jolly P. (2001). Prevalence and intensity of infections of Ascaris lumbricoides and Trichuris trichiura and associated socio-demographic variables in four rural Honduran communities. *Memórias do Instituto Oswaldo Cruz*.

[19] Mengistu E., Wossenseged L., Yeshambel B., Beyene M., Aschalew G., Belay A. (2010). Prevalence of intestinal parasites and associated risk factors among students of atse fasil general elementary school azezo, northwest Ethiopia. *Ethiopian Journal of Health and Biomedical Sciences*.

